# Spikelet movements, anther extrusion and pollen production in wheat cultivars with contrasting tendencies to cleistogamy

**DOI:** 10.1186/s12870-021-02917-7

**Published:** 2021-03-16

**Authors:** Urszula Zajączkowska, Bożena Denisow, Barbara Łotocka, Alicja Dołkin-Lewko, Monika Rakoczy-Trojanowska

**Affiliations:** 1 Department of Forest Botany, Institute of Forest Sciences, Warsaw University of Life Sciences – SGGW, 159 Nowoursynowska Street, 02-776 Warszawa, Poland; 2 Department of Botany and Plant Physiology, University of Life Sciences in Lublin, 15 Akademicka Street, 20-950 Lublin, Poland; 3 Department of Botany, Institute of Biology, Warsaw University of Life Sciences – SGGW, 159 Nowoursynowska Street, 02-776 Warszawa, Poland; 4 Department of Plant Genetics, Breeding and Biotechnology, Institute of Biology, Warsaw University of Life Sciences –SGGW, 159 Nowoursynowska Street, 02-776 Warszawa, Poland

**Keywords:** Cleistogamy, Dacanto, Flower opening, Lemma kinetics, Spikelet anatomy, Piko, Pollen dispersion, *Triticum aestivum*, Wheat breeding

## Abstract

**Background:**

Cleistogamic flowers are a main barrier in pollen dispersal for cross-pollination necessary in wheat hybrid breeding. The aim of our study was to gain new knowledge on the biology of wheat flowering, in particular on the differences between the cleisto- and chasmogamic forms which has certainly cognitive significance, but it can also be used in practice when seeking a female and male ideotypes for cross hybridization.

**Results:**

We characterized the most significant features defining the flowering specificity in two wheat cultivars with contrasting tendency to cleistogamy: Piko (chasmogamous) and Dacanto (cleistogamous). In the field observations we assessed diurnal pattern of anther extrusion and anther extrusion capacity. For the first time we adapted the time lapse method for measuring kinetics of the spikelet movement and 3-D image correlation technique for the non-invasive measurements of potential deformations of the spikelet lemmas. We found that the two cultivars differ in the potential of pollen dispersion for-cross-pollination and in the spikelet kinetics. We also described some anatomical traits that can have potential functional role in floret opening. None of the cultivars showed any symptoms of lemma surface deformation.

**Conclusions:**

The cleistogamic and chasmogamic wheat cultivars differ significantly in the potential for pollen dispersion for cross-pollination, which is mainly related to anther extrusion capacity. Although none of these features differentiated the cultivars clearly, we assume, based on spikelet kinetics and the lack of lemmas surface deformation, that the water transport and turgor of cells is essential for the floret opening and anther extrusion in wheat. The search for parental ideotype should be supported by marker assisted selection, e.g. based of polymorphisms in genes related to aquaporin biosynthesis.

**Supplementary Information:**

The online version contains supplementary material available at 10.1186/s12870-021-02917-7.

## Background

Cleistogamy is a sexual reproduction system, which is defined as the formation of closed self-pollinated flowers, has been found in many angiosperm taxa. Several plant species have mixed reproduction strategies and proportion of cleistogamous and chasmogamous flowers has been reported to be modified by environmental conditions like soil moisture, light intensity, fertilization of soil and plant density [1]. Hexaploid common wheat (*Triticum aestivum*), which is one of the most important world food plant, is a self-pollinated species with mostly cleistogamous florets adapted for self-pollination and fertilization in closed florets [2–4]. Cleistogamy is the main disadvantage for hybrid breeding [5–8]. The male components of cross hybridization, regardless of the mechanism on which it is based – natural (e.g. *cms-Rf*) or artificial (chemical emasculation) - should have chasmogamous flowers that are open at anthesis which facilitate anther extrusion and, consequently, pollen shedding for cross-pollination [9–12]. Wheat florets are arranged in a spike inflorescence, typical for Poaceae (Gramineae). The spike main axis bear spikelets. There are two glumes at the base of each spikelet. Each spikelet consists of two-five florets. A floret is perfect and consists of a lemma and palea, three stamens, one pistil, and two lodicules located between the lemma and the ovary base [2, 13, 14]. The floret opening at anthesis in cereals is thought to be due to the lodicules swelling [2, 15–18]. In wheat, the pair of lodicules located between the lemma and the ovary base expand rapidly at the time of anthesis, push away the rigid lemma allowing anthers and stigma to emerge [16, 19]. The process induced by the turgid lodicules called as ‘first opening’ is rather short and usually lasts for less than 30 min [2, 20, 21]. The ‘second opening’ of wheat floret was recently described and the authors suggested that unfertilized ovary increase in radial dimensions and generates lateral push of the rigid lemma and palea [22]. However, it is still not clear whether during the ‘first opening’ the lemma undergoes deformations, which could increase the efficiency of floret opening and anther extrusion. Also, the kinetics of the floret opening has so far been poorly studied. It is known that plants have evolved various mechanisms of movement of certain morphological structures that are important in reproduction processes, such as floret opening, pollen dispersion or seed distribution [23, 24]. In many cases of ferns and flowering plants, the energy that activates the onset of movement is due to the uneven cell wall thickness or the anisotropy of a specific structure that is deformed in the evaporation process [25, 26]. To date, however, there has been no experimental data on the possibility of lemma deformation during rapid expansion of swelling lodicules and ovary base that occur at the time of the floret opening at anthesis in cereals. There is also no data on the kinetics of the flower opening process. Pollen production is also among the traits reported to be important in hybrid wheat breeding [4, 10, 27, 28]. However, most cultivated forms of common wheat produce low amounts of pollen, which is indicated as a significant limitation in wheat hybrid breeding programs [7, 11, 29].

In this study, we surveyed the kinetics and the micromorphological and anatomical structures of spikelets in two wheat cultivars with contrasting tendency to cleistogamy. Two winter wheat cultivars - Piko (chasmogamous) and Dacanto (cleistogamous) were used. For the first time we applied the time lapse technique for measuring the kinetics of spikelets movements and the non-invasive measurements of potential deformations of the spikelet lemmas during anthesis. Moreover, we analyzed if the structural features and changes in the pre-anthesis and post-anthesis spikelets can determine the anther extrusion ability. In addition, we tried to assess to what degree the floral biology (diurnal pattern of anther extrusion, the efficiency for anther extrusion, the pollen production and pollen dispersion capability) differ between two contrasting cultivars.

## Results

### Anther extrusion

**Fig. 1:**
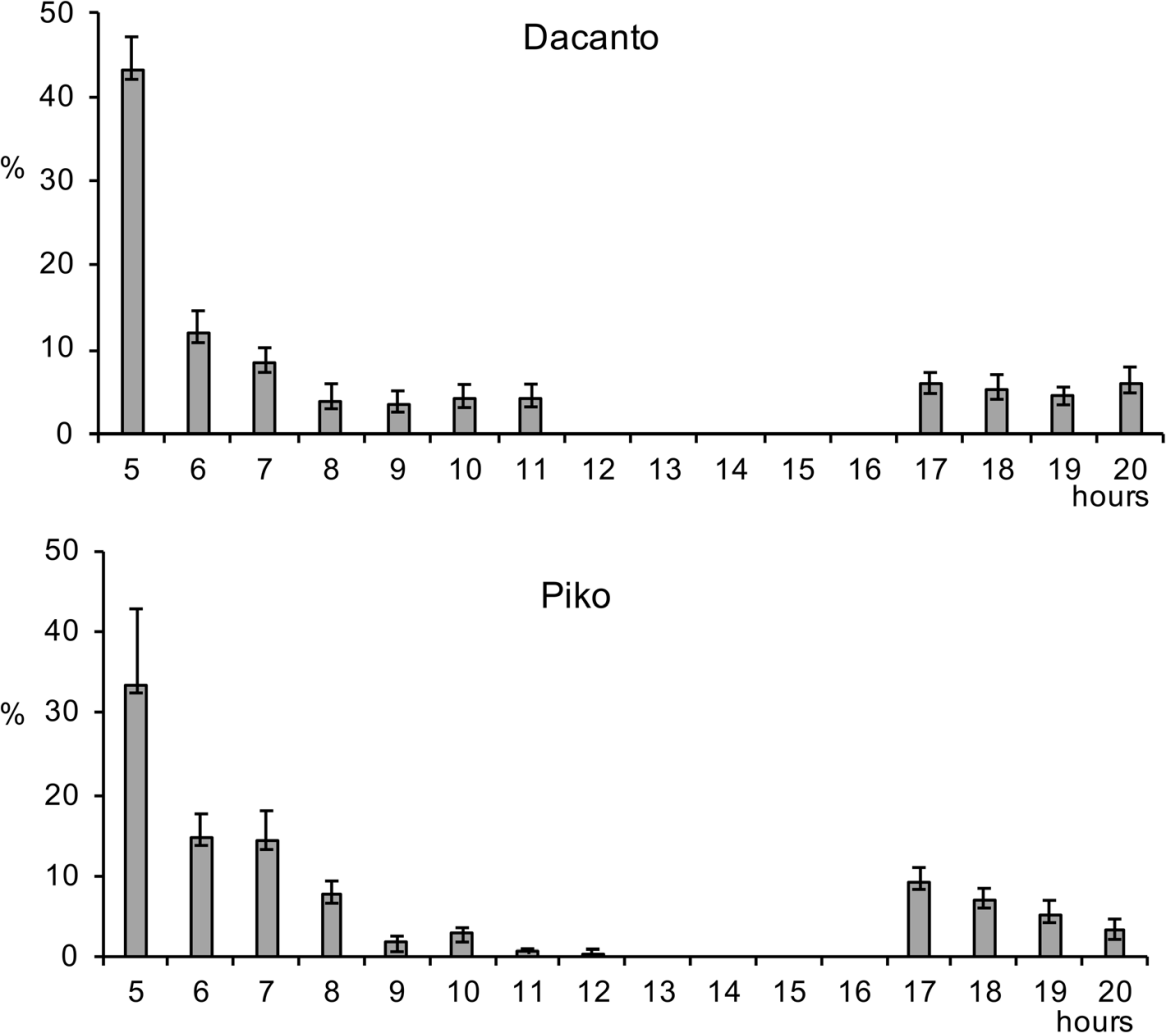
Diurnal pattern of anther extrusion in two wheat cultivars. The extrusion is expressed as the number of anthers extruded at one hour intervals (GMT + 2 h) in relation of the total number of anthers extruded per day (average from 2018 to 2019). Whiskers indicate ± SD; hours GMT + 2 h.

**Fig. 2:**
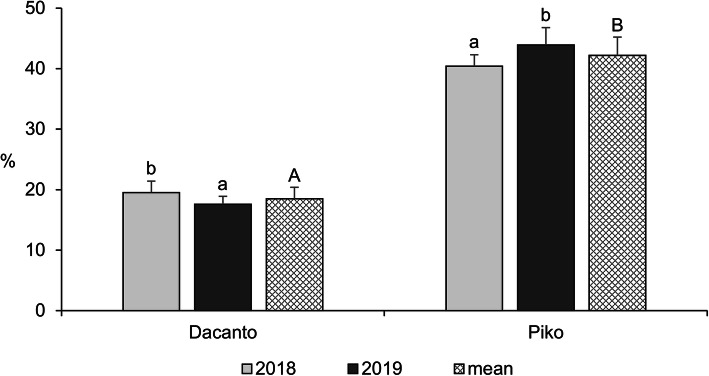
Proportion of extruded anthers in two wheat cultivars observed in 2018 and 2019. Untransformed data are presented. Whiskers indicate ± SD. Means followed by the same small letter are significantly different between the years and those followed by the same capital letter - between the cultivars at *P* < 0.05, according to the Mann-Whitney’s U test

The diurnal pattern of anther extrusion was similar in both genotypes grown in the field conditions (Fig. 1, Additional file 1: Table S1). The peak of anther extrusion was observed in early morning hours (between 5.00 h and 8.00 h). A total lack of anther extrusion was characteristic for mid-day hours. Towards the end of the day, during evening hours, a small proportion of anthers have been extruded (approximately 3–5% of the daily amount in Dacanto and 10% in Piko). The number of anthers per spike was statistically non-significant between cultivars (Mann-Whitney U test: Z = − 0.65, *P* = 0.516; Table 1, Additional file 1: Tables S3-S4). Considerable year-to-year differences were documented for the number of anthers developed in spikes (for cv. Dacanto *Z* = 3.74, *P* = 0.000; for cv. Piko *Z* = 3.71 *P* = 0.000) and lower values were established in 2019 compared to 2018. The proportion of extruded anthers differed considerably between the cultivars (*Z* = − 5.39, *P* = 0.000; Fig. 2, Additional file 1: Table S2. Approximately two-fold higher proportion of extruded anthers was noted for the cv. Piko compared to the cv. Dacanto (42.2% ± 1.9 vs. 18.6% ± 3.0, respectively). The year effect was found within cultivars for the ability to anther extrusion (for cv. Dacanto *Z* = 1.96, *P* = 0.049; for cv. Piko *Z* = − 2.88, *P* = 0.004).

**Table 1:** Components of pollen production in two wheat cultivars across years of study

Cultivar	Years	Number of flowers per spikelet		Number of spikelets per spike		Number of flowers per spike		Number of anthers per spike		Number of pollen grains per anther	
		mean	±SD	mean	±SD	mean	±SD	mean	±SD	mean	±SD
Dacanto	2018	4.5_b_	1.7	18.8_a_	1.7	84.4_b_	7.8	253.1_b_	23.0	923.0_a_	418.4
	2019	2.7_a_	1.2	19.3_a_	1.4	53.0_a_	3.8	159.5_a_	11.7	1850.5_b_	583.5
	Mean	3.6_A_	1.8	19.1_A_	1.6	68.7_A_	16.8	206.3_A_	50.8	1386.8_B_	640.3
Piko	2018	4.0_b_	0.9	21.5_a_	1,0	86.0_b_	4.0	258.0_b_	12.0	885.3_a_	379.5
	2019	2.8a	1.2	20.3_a_	1.3	56.7_a_	3.7	169.0_a_	11.1	1600.6_b_	282.1
	Mean	3.4_A_	1.2	20.9_A_	1.3	71.35_A_	15.5	213.5_A_	46.6	1243.0_A_	482.3

Means followed by the same small letter are not significantly different between years and that followed by the same capital letters between cultivars at α =0.05

### Pollen production

**Fig. 3:**
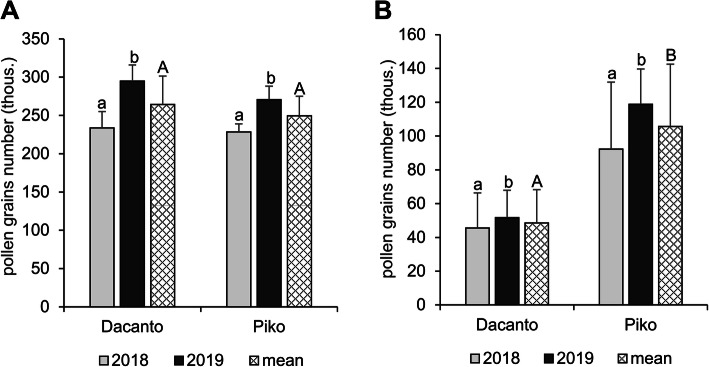
Total pollen production per spike (**A**) and pollen dispersion capacity per spike (**B**). Results of the experiments with two wheat cultivars performed in 2018 and 2019. Whiskers indicate ± SD. Means followed by the same small letter are not significantly different between the years and those followed by the same capital letter - between the cultivars at *P* < 0.05, according to t-test

The number of pollen grains produced per anther differed significantly between the cultivars (*t* = 2.00, df = 406, *P* = 0.000; Table 1) and was higher in the cv. Dacanto. A year-to-year disparity was found in the amount of pollen in the anthers (Dacanto *t* = −11.79, df = 202, *P* = 0.000; Piko *t* = −13.43, df = 202, *P* = 0.000). In both cultivars, a statistically higher amount of pollen was produced in 2019 (ca. 2-fold higher than in 2018). The total pollen production per spike was similar in both cultivars (*t* = 1.73, df = 78, *P* = 0.087; Fig. 3A, Additional file 1: Table S4). However, a year effect on the number of pollen grains produced in spikes was found (Dacanto *t* = − 7.58, df = 38, *P* = 0.000; Piko *t =* − 5.00, df = 38, *P* = 0.004). The cultivars differed significantly in the number of pollen grains released for cross-pollination (*t* = − 21.15, df = 78, *P* = 0.000), and the cv. Piko exhibited a higher amount of dispersed pollen (Fig. 3B, Additional file 1: Table S4). The number of pollen grains available for cross-pollination differed between the seasons (Dacanto *t* = − 4.24, df = 38, *P* = 0.000; Piko *t =* − 7.58, df = 38, *P* = 0.000).

### Micromorphology and anatomy of spikelets

**Fig. 4:**
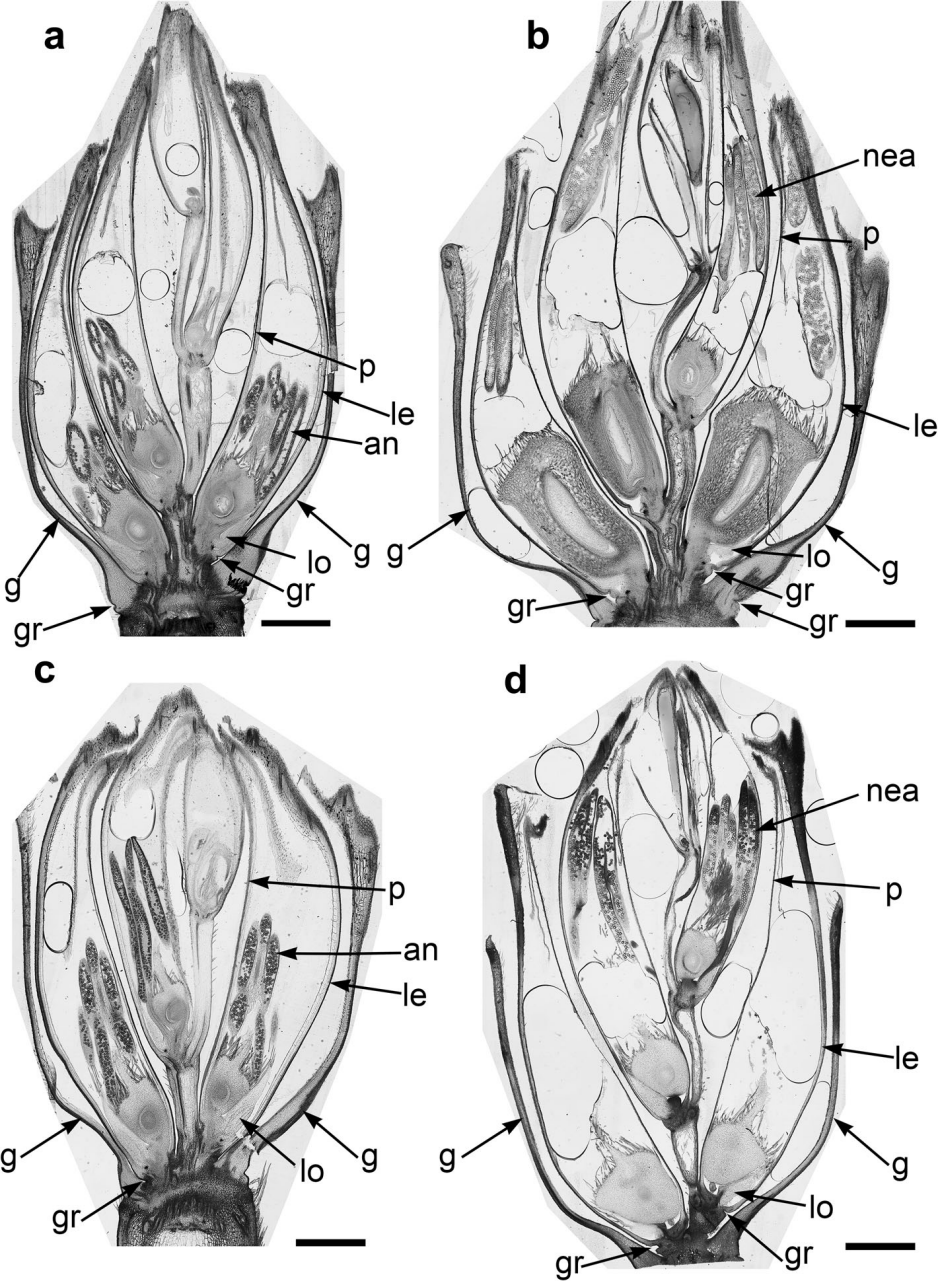
General anatomy of the spikelet in cv. Dacanto (**a**, **b**) and cv. Piko (**c**, **d**). Spikelets before anthesis (**a**, **c**) and after anther extrusion (**b**, **d**). Arrows, g- glume, le- lemma, p- palea, lo-lodicule, an-anthers, nea- non-extruded anthers, gr - grooves. Scale bars = 1500 μm

**Fig. 5:**
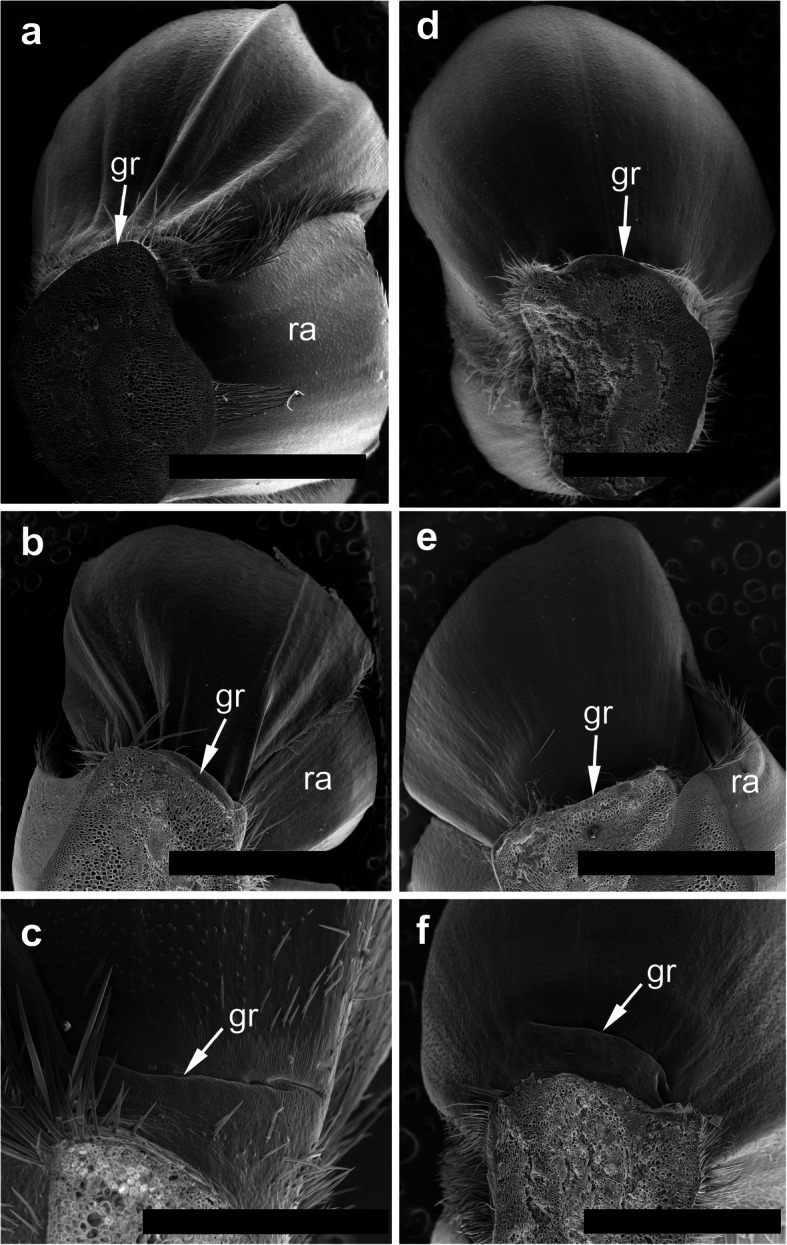
Micromorphology of glumes (**a-c**) and lemmas (**d-f**). Spikelets of cv. Dacanto (**a**, **b**, **d**, **e**) and cv. Piko (**c**, **f**) before anthesis (**a**, **c**, **d**, **f**) and after anther extrusion (**b**, **e**). Small grooves (gr) are visible in the basal parts of the glumes and lemmas already before anthesis, ra – rachilla. Scale bars in (**a**), (**b**), (**d**), (**e**) = 2000 μm, bar in (**c**) = 500 μm and bar in (**f**) = 1000 μm

**Fig. 6:**
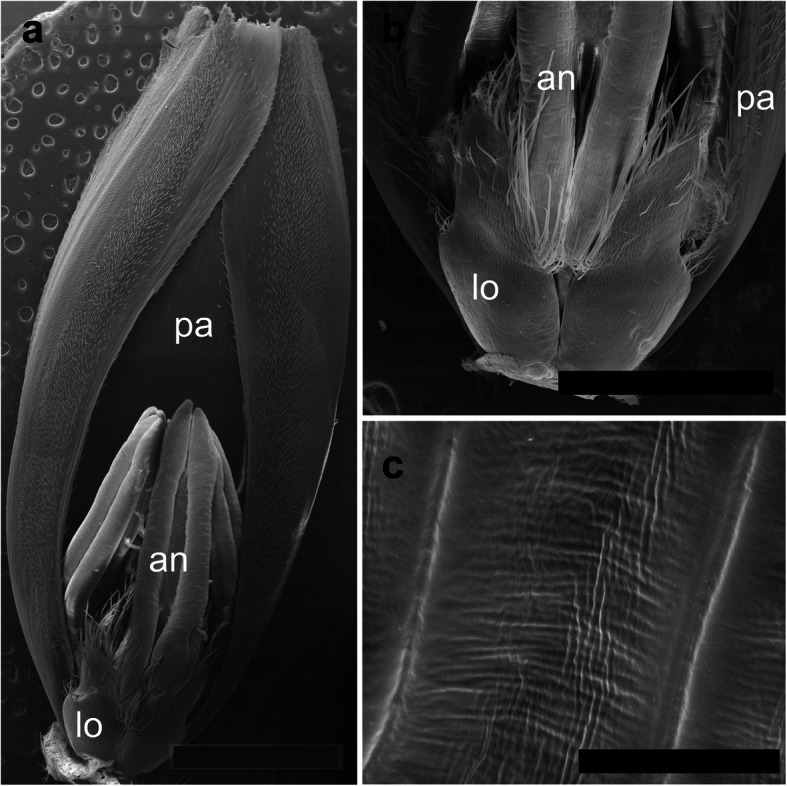
Micromorphology of cv. Dacanto spikelet before anthesis: palea (cut apically) with a floret attached (**a**). Lodicules of cv. Piko before anthesis (**b**); a criss-cross pattern of the lodicule cell surface in cv. Dacanto (**c**); pa – palea, lo – lodicules, an – anthers. Scale bar in (**a**) = 2000 μm, bar in (**b**) = 1000 μm, and bars in (**c**) =100 μm

The spikelets of both cultivars consisted of two glumes and 4–6 florets, including 3 fertile ones in cv. Dacanto (Fig. 4a, b), or 4–5 florets, including 2–3 fertile ones in cv. Piko (Fig. 4c, d) set distichously and alternately on the rachilla, with antherns positioned higher within the florets in Piko. Characteristically, in every sectioned post-anthesis spikelet of the cv. Dacanto, all florets contained some non-extruded anther(s) (Fig. 4b). In the spikelets of the cv. Piko examined post anthesis, all anthers were absent at least in the basal two florets (Fig. 4d). In the same spikelets, the upper 1 or 2 florets retained stamens within the lemma and palea, and their filaments were slightly or non-elongated. The glumes were boat-shaped, with a massive base in both cultivars (Fig. 5a-c). In this area, the glume abaxial epidermis in both cultivars was smooth and composed of elongated non-turgid pavement cells. In this domain, a minute and shallow transversal groove was discernible before anthesis (Fig. 5a, c). In spikelets fixed post-anthesis, widening of the grooves within the bases of glumes was visible (Fig. 5b). In both cultivars, the two basal florets formed the largest pistils with a well-formed gametophyte. The third flower was smaller but properly built as well, and the distal 2–3 florets were minute, apparently aborted at the early developmental stage (Fig. 4a, b). Each floret was supported by a lemma and palea. The lemmas were boat-shaped, like the glumes, but wider and longer (Fig. 5d-f). Like in the glumes, a groove was discernible at pre-anthesis also in the lemma bases within a domain of non-turgid pavement cells of abaxial epidermis. The two lodicules were both facing lemma. Due to such location, in the dorsoventral longitudinal sections (Fig. 4a-d) only a single lodicule was visible in every fertile floret. At pre-anthesis, lodicules were turgid, with basal half convex and the apical one rather flat and covered with long, thick-walled and spiked trichomes (Fig. 6a, b). The epidermis of lodicules was composed exclusively of rather short pavement cells, with straight anticlinal walls and characteristic surface microsculpture (Fig. 6c). Post anthesis, the lodicules were turgor-less and crushed (Fig. 4b, d).

### Lemma kinetics

**Fig. 7:**
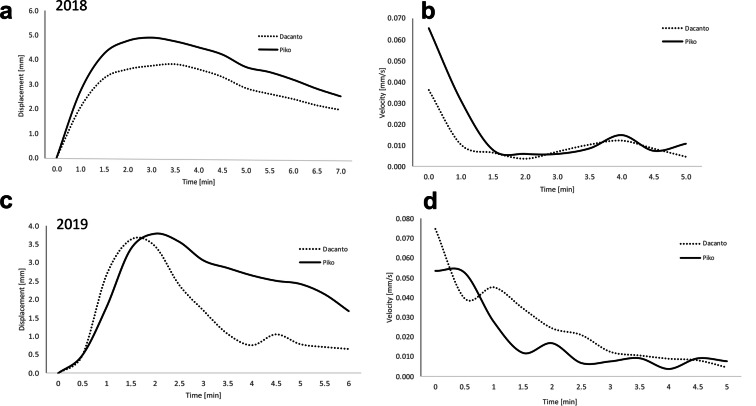
Spatial displacement (**a**, **b**) and speed of movement (**c**, **d**) of the opening lemmas. Results of two experiments during flowering of Piko and Dacanto wheat cultivars. Carried out in 2018 (**a**, **c**) and 2019 (**b**, **d**). Data obtained from the time-lapse imaging transformed in the Tracker program


The analysis of time-lapse movies shows that lemma opening period lasted on average about 5 min, and for individuals it ranged from 2 to 15 min (Fig. 7, Additional file 1: Table 5). The maximum lemma opening was on average about 1.5 min from the beginning of the process in the 2018 experiment and about 1.5–2 min in 2019. During this first period of flowering, the highest speed of lemmas displacement was also noted. Comparison of lemmas displacement kinetics shows that wider lemmas opening occurs in the Piko cultivar. In 2018 this was observed for almost the entire lemma opening period and in 2019 it was revealed in the period after the maximum opening time, when the degree of lemma opening was gradually decreasing. Additional movie files illustrate the original time-lapse movies recorded during the lemmas opening in two cultivars (see Additional files 2 and 3).

**Additional file 2: Movie 1.** Example of the original time-lapse movie recorded during the lemmas opening in cv. Dacanto.


**Additional file 3: Movie 2.** Example of the original time-lapse movie recorded during the lemmas opening in cv. Piko.

**Fig. 8:**
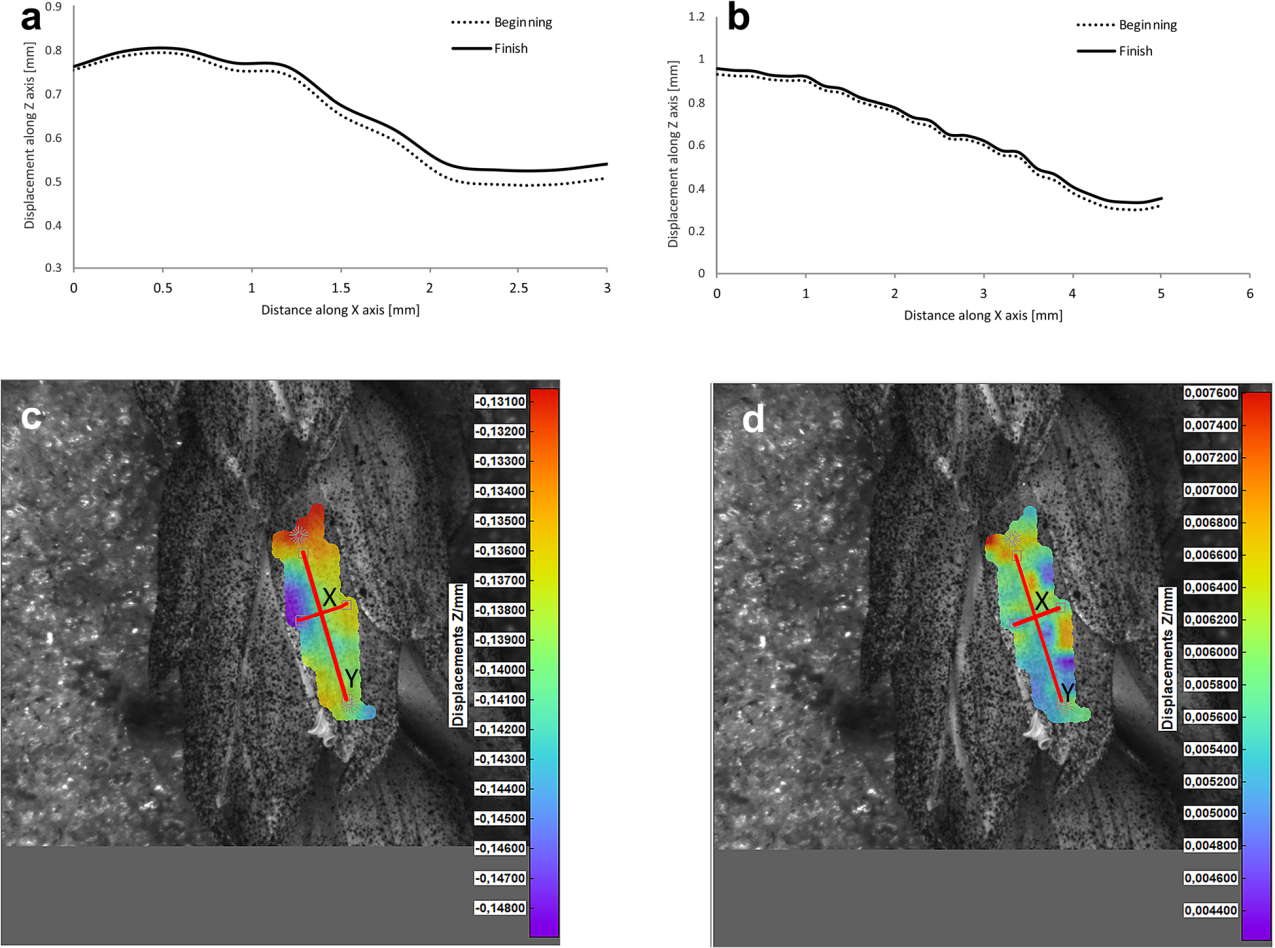
Results of the lemma surface deformation test using the 3D image correlation method. Diagrams (**a**, **b**) and maps (**c**, **d**) of the lemma surface displacement. The graphs presented in (**a**) and (**b**) show the Z values of the points on the lemma surface along X and Y axes, respectively, at the beginning of the flowering process before the lemma opening (**c**) and after the first phase of opening (**d**). Piko cultivar, lemma No. 1

**Fig. 9:**
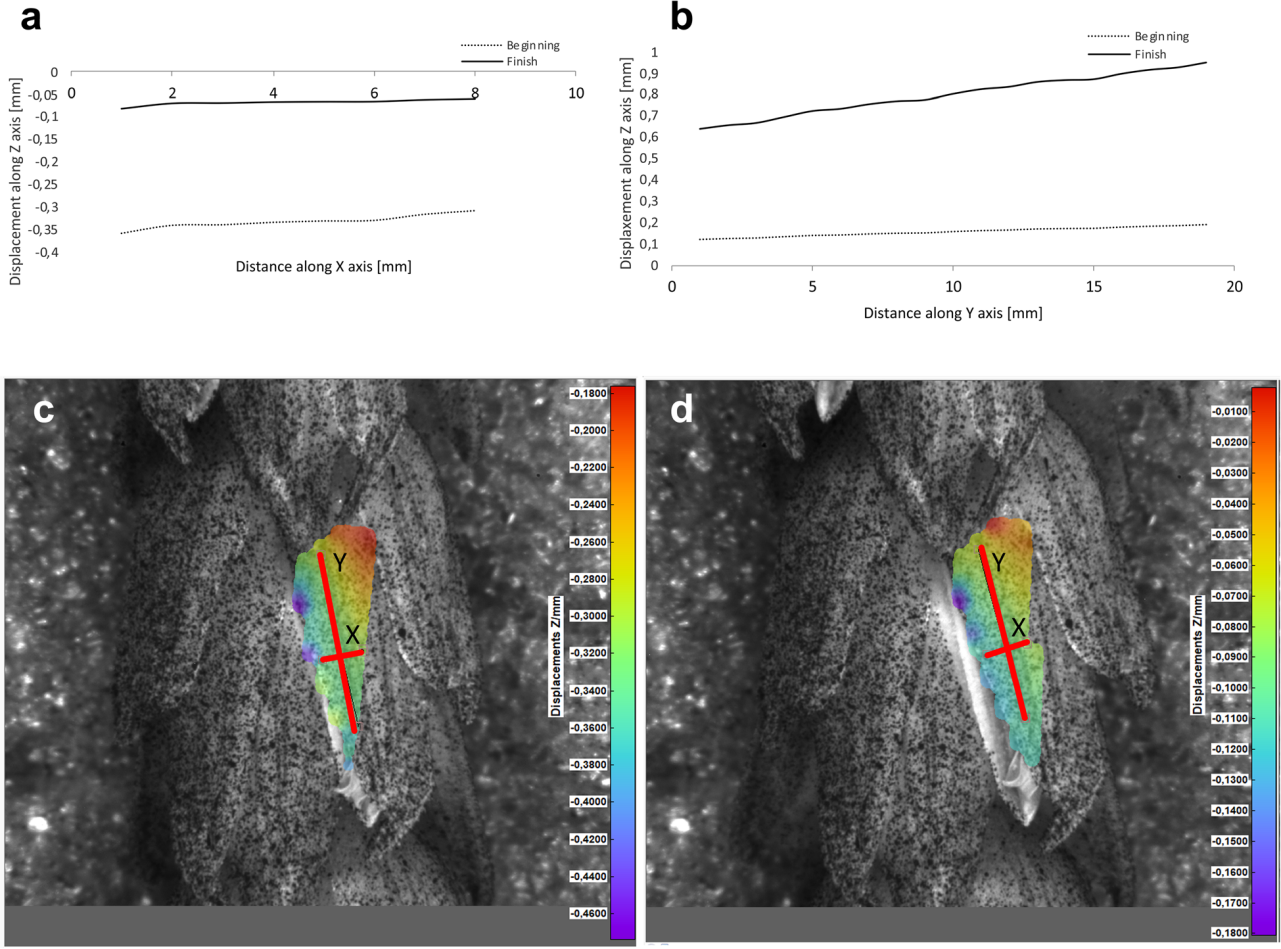
Results of the lemma surface deformation test using the 3D image correlation method. Diagrams (**a**, **b**) and maps (**c**, **d**) of the lemma surface displacement. The graphs presented in (**a**) and (**b**) show the Z values of the points on the lemma surface along X and Y axes, respectively, at the beginning of the flowering process before the lemma opening (**c**) and after the first phase of opening (**d**). Piko cultivar, lemma No. 2

**Fig. 10:**
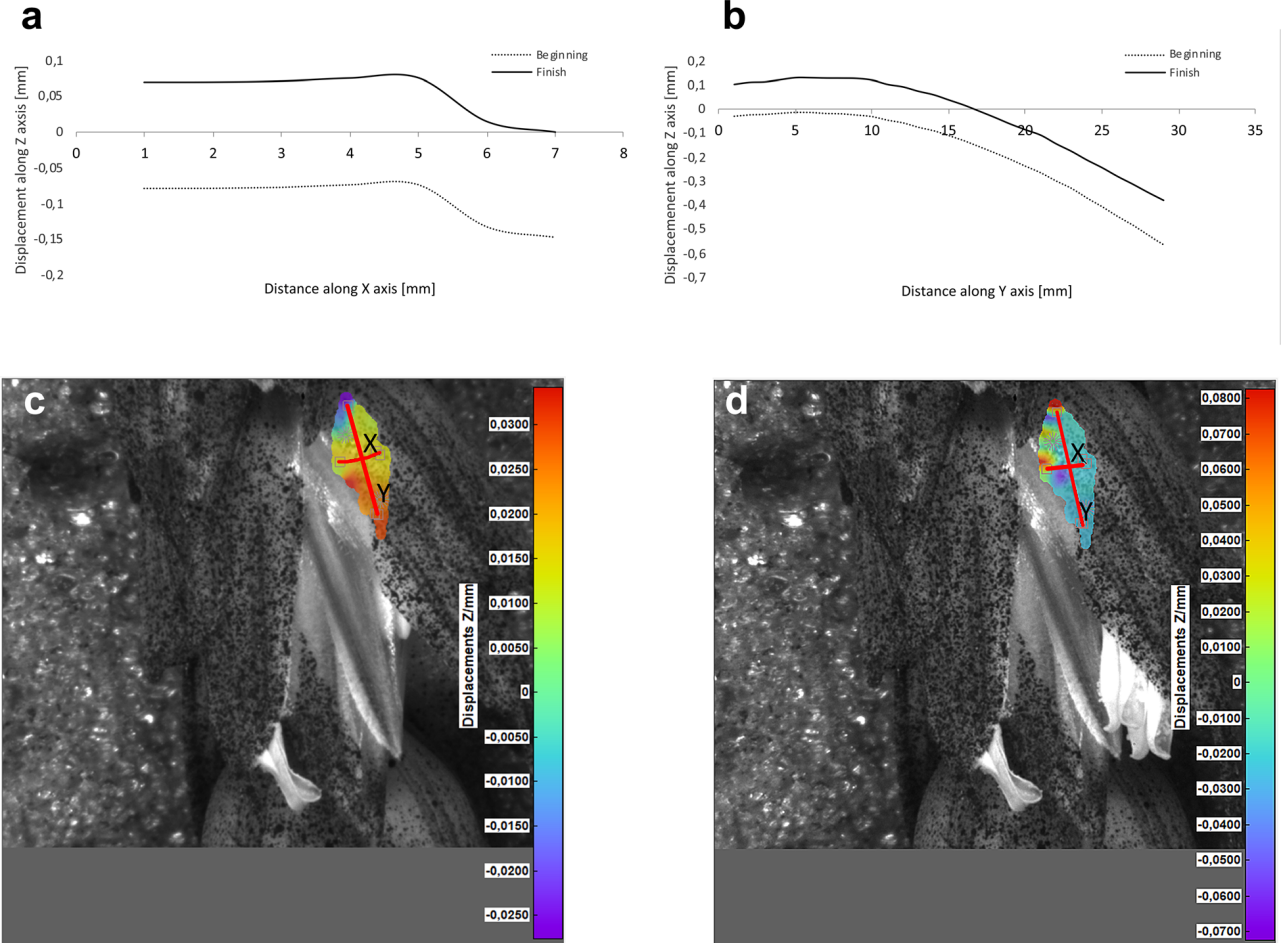
Results of the lemma surface deformation test using the 3D image correlation method. Diagrams (**a**, **b**) and maps (**c**, **d**) of the lemma surface displacement. The graphs presented in (**a**) and (**b**) show the Z values of the points on the lemma surface along X and Y axes, respectively, at the beginning of the flowering process before the lemma opening (**c**) and after the first phase of opening (**d**). Dacanto cultivar, lemma No.1

**Fig. 11:**
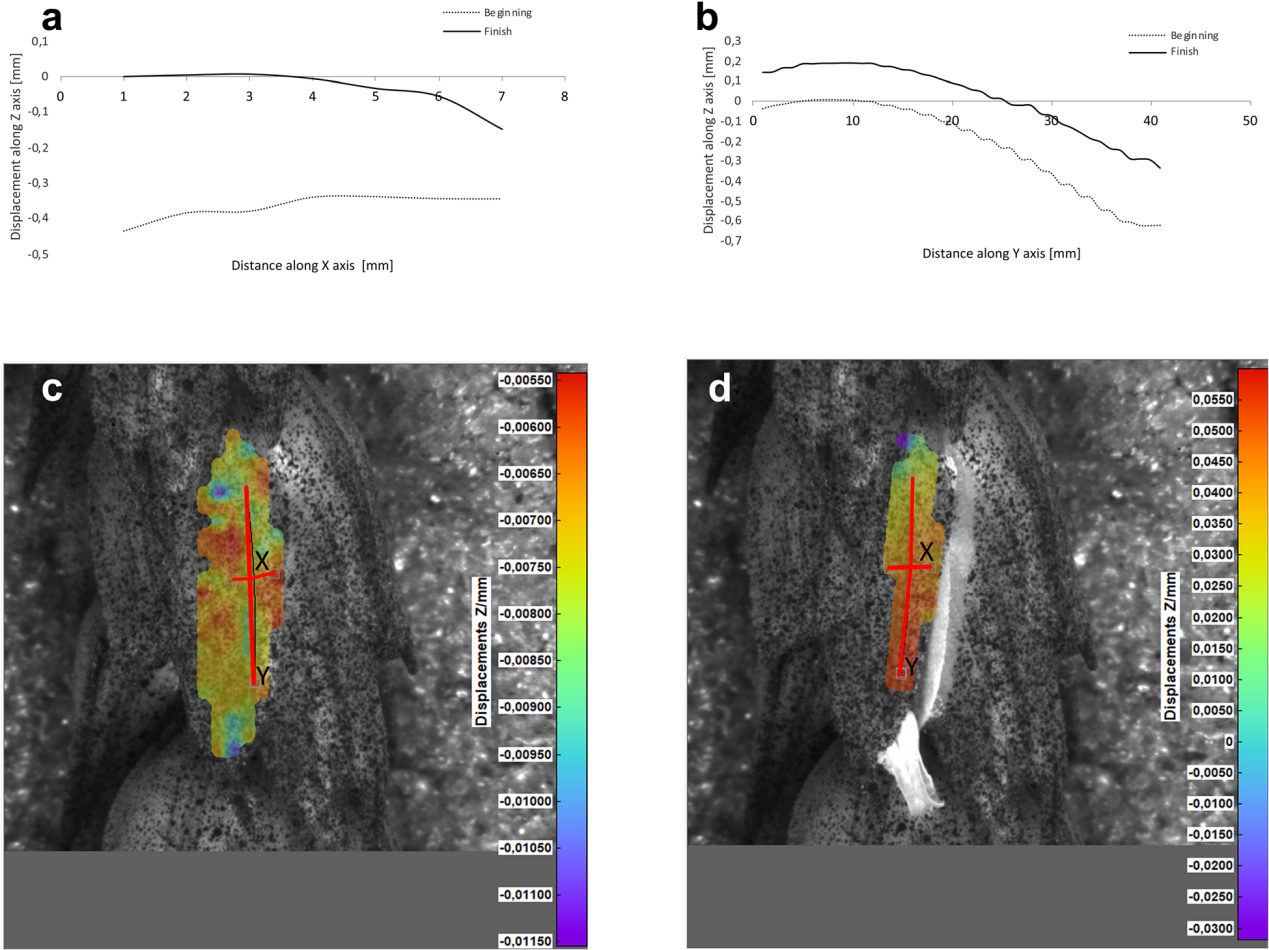
Results of the lemma surface deformation test using the 3D image correlation method. Diagrams (**a**, **b**) and maps (**c**, **d**) of the lemma surface displacement. The graphs presented in (**a**) and (**b**) show the Z values of the points on the lemma surface along X and Y axes, respectively, at the beginning of the flowering process before the lemma opening (**c**) and after the first phase of opening (**d**). Dacanto cultivar, lemma No.2

Potential deformations of lemmas during the flowering process was measured using a 3-D image correlation method along two rectangular X-Y axes (Figs. 8, 9, 10, 11). The longer Y axis was parallel to the lemma venation pattern and the X axis was oriented perpendicularly. The color maps presented in Figs. 8 c,d, 9, 10 and 11c,d show the Z values of the points on the whole lemma surface at the beginning of flowering process before the lemma opening and at the initial phase after opening. The end of the measurement period was limited by the depth of field of the lens of the stereoscopic microscope and occurred when during the lemma displacement the surface of the lemma became out of focus of the lens. A detailed analysis of the potential lemma surface deformation can be done on the basis of the data shown in the graphs in Figs. 8a,b, 9, 10 and 11a,b where the Z values of the points along X and Y axes are presented. The comparison of the pattern of the graphs for the same lemma at the beginning and the end of measurement indicate that during initiation of the lemma opening there is a spatial position shift of whole lemma surface without any noticeable displacement between the reference points distributed within the lemma surface which suggests that there are no symptoms of the lemma surface shape deformation.

An additional movie file illustrates, as an example, the change in the position of the lemma during flowering, recorded from two cameras connected to a stereoscopic microscope, which were the basis for determining the potential lemma deformation using 3-D image correlation method (see Additional file 4).


**Additional file 4: Movie 3.** Movie illustrating, the change in the position of the lemma during flowering. Original records from two cameras connected to a stereoscopic microscope, which were the basis for determining the potential lemma deformation using 3-D image correlation method.

## Discussion

In this study we presented a detailed comparative characteristics of flowering biology of two contrasting winter wheat cultivars – cleisto- and chasmogamic, which comprised morpho-anatomical study of spikelet structure including their kinetics, anther extrusion and pollen production. Such a complex study employing different analytical approaches was performed in wheat for the first time. Our anatomical observations revealed that in every sectioned post-anthesis spikelet of cv. Dacanto, all florets contained some non-extruded anther(s), while in cv. Piko spikelets nearly all anthers were absent at least in the basal two florets. These findings correspond to field observations that exhibited two-fold higher proportion of anthers extruded from the Piko spikes.

Microscopic analysis showed that spikelets anatomy of the two cultivars, in general is similar to that reported in classical papers concerning floret anatomy of the bread wheat genotypes [13, 30]. However, certain details of the spikelet micromorphology were not reported earlier. This novel trait that we consider as having potentially functional role in floret opening concerns formation of grooves within the glume and lemma bases and deepening of the grooves during anthesis. Although the grooves seem more pronounced (wider) in cv. Piko, which is a better extruder of anthers, from the structural point of view both cultivars can potentially extrude anthers with similar efficiency. As it is seemingly not the case, the reasons should be sought in the functioning of the spikelet elements, especially in the water transport and turgor control [19]. This suggestion is confirmed by our results of measuring the kinetics of lemmas openings in which for the first time the time lapse technique was applied. The measurements showed that the average length of this period is about 6–7 min, and for individual spikelets it is in the range of 2–15 min. The data are consistent with the reports of earlier authors indicating that the actual opening of the floret in most grasses is caused by the lodicules becoming suddenly swollen at the base, so the lemma is levered outwards [22]. The difference between the two cultivars is, however, small, but the kinetics parameters seem to be more favorable for anther extrusion and to the cross- pollination processes for the cv. Piko.

The diurnal pattern of anther extrusion can also support the importance of hydration of spikelet elements for the anther extrusion process. The peak of anther release was observed in early morning hours, when the plant cells are usually well hydrated [24]. A fundamental role of the water balance, the turgor and its regulation in flower opening have been confirmed in many studies [31]. The key role of water transport and turgor of cells in lodicules is also confirmed by the results of our studies on the possible deformations of lemmas in the process of florets opening using the non-invasive 3-D image correlation technique, which was for the first time applied to plant biological material. Due to the limited range of depth of field of camera lenses, these studies were limited to the initial lemmas opening period only, however this period seems to be crucial for the process and the results obtained can be considered to be reliable for the whole process. The clearly demonstrated for the first time the absence of any deformation of the lemma surface indicates that in the process of wheat florets opening, the potential mechanical effects associated with lemmas strains do not play a significant role, and the mechanisms related to cell turgor and water transport to lodicules should be the crucial factor. These results may suggest that in future studies on mechanisms associated with wheat flowering biology, more attention should be focused on aquaporins. Genes encoding aquaporins in wheat have been already recognized, e.g. Pandey et al. [32] indicated 13 wheat aquaporin genes, including genes coding for tonoplast aquaporins. Despite transcripts of two genes (PIP1–2 and TIP2–1, encoding the plasma membrane intrinsic proteins and tonoplast intrinsic proteins, respectively) were detected at a relatively high level in florets and inflorescences, the authors, however did not indicate the clear role of any of these genes in the processes related to wheat floret opening.

Our survey has shown that the wheat cultivars with contrasting tendency to cleistogamy differ in the potential for pollen dispersion. The cultivar Dacanto with higher pollen production per anther and similar pollen production per spike compared to Piko, has displayed lower amount of dispersed pollen (i.e. the number of pollen grains available for cross-pollination), therefore we conclude that the anther extrusion capability is particularly important for the provision of air-blown pollen for cross-pollination in wheat. Several studies have reported that the ability for anther presentation outside the floret is essential for pollen dispersal in wheat [4, 7, 10, 29]. However, the number of pollen grains production per anther can not be ignored, as indicated by de Vries [27], who pointed out that pollen donor capacity is positively related to the total number of produced pollen grains. In our study, the number of pollen grains established per anther (averaged 1243–1386) is within that reported in literature for wheat cultivars, e.g. 975 to 2773 [33]. However, our values recognized for cv. Piko were lower than that reported for this cultivar by de Vries [27], who documented 2672–2933 of pollen grains per anther. These disparity can be understood as pollen production even in this same species/cultivars is sensitive to external factors and can vary greatly spatially and temporally [34, 35]. The differences could be the results of the human activities, i.e. fertilization, irrigation or their combination. It was reported in a number of studies that decrease in nutrient supply may result in the reduction of pollen grains formed per anther [36, 37]. Likewise the method used for the assessment of pollen grains number can impact on the records.

Similarly, the temporal (=between years) differences established in pollen production (per anthers, per spikes) in both cultivars can be associated, at least partially, with specific environmental factors occurring during pollen development. Every stages of pollen development, i.e. the differentiation of sporogenous cells and meiosis (microsporogenesis) as well as post-meiotic development of microspores are recognized as the most stress-sensitive period in plant reproduction [36]. The role of drought stress, moderate water–deficit stress, suboptimal temperature and/or oxidative stresses on pollen production has been demonstrated across wheat cultivars [3, 38]. According to Monneveux et al. [39], pollen development in wheat is critically affected by imbalance between photosynthesis and translocation use of photoassimilates (mainly starch). Year-to-year disparity in the pollen production calculated for spikes can be associated with both the differences in the pollen production per anther and the variable number of anthers developed in inflorescences across growing seasons. Such relationships have been previously documented in many plants [34, 35].

## Conclusions

The aim of our study was to gain new knowledge on the biology of wheat flowering, in particular on the differences between the cleisto- and chasmogamic forms which has certainly cognitive significance, but it can also be used in practice when seeking a female and male ideotypes for cross hybridization. In conclusion, the cleistogamic and chasmogamic wheat cultivars differ significantly in the potential for pollen dispersion for cross-pollination, which is mainly related to anther extrusion capacity. We described several novel anatomical traits that can have potential functional role in floret opening. Although none of these features differentiated the cultivars clearly, we assume, based on spikelet kinetics and the lack of lemmas surface deformation, that the water transport and turgor of cells is essential for the floret opening and anther extrusion in wheat. The search for parental ideotype should be supported by marker assisted selection, e.g. based of polymorphisms in genes related to aquaporin biosynthesis.

## Materials and methods

### Plant material

Two winter wheat cultivars - Piko (chasmogamous, bred by Nordsaat Saatzucht GmbH, Germany), and Dacanto (cleistogamous, bred by KWS Polska Sp. z o.o., Poland) were used.

For the field study on the floral biology the experimental plants were grown under field conditions at the University of Life Sciences (ULS) in Lublin, Poland (51° 14′ N, 22° 34′ E, altitude 238 m a.s.l) in 2018–2019. The soil is podzolic, developed from loess. The soil reaction pH in H_2_O ranged 5.8–6.8. The average concetrations of nutriens in soil at depth of 0–20 cm is presented in Table 2. The plants used for anatomical study were grown in the collection of agricultural crop plants (Department of Agronomy) at the Warsaw University of Life Sciences (WULS) campus in Ursynów, Warsaw (52° 16′ N, 21° 05′ E) in 2018. Plants were cultivated in podzolic loam soil at pH 6–7. Caryopses of each cultivar were sown in September (Warsaw) or October (Lublin), in plots (*n* = 5) 4.0 m long. From each plot 10 plants were collected for anatomical studies. No fertilization was applied. Standard procedures for winter wheat plant protection were applied. Weeds were removed by hand, if necessary.

**Table 2:** Content of nutrient elements in the arable layer (0–20 cm) of the soil in the experimental plot

Nutrient (mg/1 dm^3^ of soil)					
N0_3_^−^	P	K	Ca	Mg	Cl^−^
9.3	44.0	27.5	473.7	111.5	13.1

### Field study

The field observations were conducted to assess (i) diurnal pattern of anther extrusion and (ii) anther extrusion capacity. The diurnal pattern of anther extrusion was exhibited based on methods described by Langer et al. [4] and was recorded between 4.00 h (GMT + 2 h) and 20.00 h, in one-hour intervals, using *n* = 10 spikes per cultivar. Diurnal pattern of anther extrusion was expressed as the percentage of newly extruded anthers in relation to the total number of anthers extruded during a day. These observations were conducted in full flowering phase of each cultivar. The anther extrusion capacity was determined on the basis of the proportion of anthers released out of florets in relation to all anthers developed per spike. To establish this, we covered spikes (*n* = 20 per cultivar per year) with isolators (mesh size < 1 mm) before flowering and counted the number of released anthers as well as the number of all developed anthers. Isolators were kept until the end of the spike flowering period.

### Pollen dispersed for cross-pollination

Pollen production was evaluated as (i) total pollen production, i.e. the number of pollen grains produced per spike, and (ii) capability for pollen dispersion, i.e. the number of pollen grains dispersed outside the spike. The capability for pollen dispersion was calculated based on total pollen production and the anther extrusion capacity (for definition see Field study subsection).

The number of pollen grains produced per spike was calculated based on the number of anthers developed per spike (*n* = 20 spikes per cultivar per year, collected from different plants) and the number of pollen grains produced per anther. The number of pollen grains per anther was established using electronic particle counter (Multisizer 4e, Beckman Coulter Counter, Inc., USA) calibrated according to the producer recommendations. The pollen grains were counted in mature anthers collected just before opening. Anthers were dissected from florets randomly selected form different spikes. In total, 408 anthers were sampled (*n* = 204 for Dacanto and for Piko). Anthers were placed in Eppendorf Tubes 2.0 ml. Subsequently, pollen was removed from anthers using ether (2 times × 1 ml) and 70% ethanol (5–7 times × 1–2 ml). Finally, ethanol was evaporated, the electrolyte was added and counting of pollen grains was made automatically using an electronic sensor.

### Micromorphology and anatomical study

Entire inflorescences (spikes) were taken for microscopic examination, in two terms (i) before (= pre-anthesis) and (ii) after opening (= post-anthesis) the lemmas. The inflorescences were fixed in pure methanol overnight [40], and next rinsed a few times (and stored) in absolute ethanol. During rinsing, sterile basal spikelets and the basal fertile spikelet were removed, and the next 3–4 fertile spikelets were later excised for light microscopy (LM), and the next ones for scanning electron microscopy (SEM).

Spikelets intended for LM were stained whole prior to embedding with the safranin-Astra blue (S-AB; Maácz and Vágás [41]). The most uniform staining was achieved in rehydrated spikelets, with the staining mixture slowly instilled into the spikelet using a syringe. Stained spikelets were rinsed a few times with 70% ethanol and embedded for vibratome sectioning. Since the blade pulled the spikelets from the 7% agarose blocks, the specimens were embedded in Glycid Ether 100 Epoxy Resin (SERVA) grade very soft (without the hardener, prepared according to the manufacturer’s formula), as follows (i) dehydration in graded ethanol series, with each stage prolonged to 30 min and overnight final dehydration in absolute ethanol, (ii) propylene oxide, (iii) saturation in resin-propylene oxide mixtures with each stage prolonged until sinking of most specimens, (iv) overnight saturation with pure resin and placement of samples in flat embedding molds for sectioning at the ventral-dorsal longitudinal plane, (v) preliminary polymerization at room temperature overnight to facilitate evaporation of propylene oxide residues (this stage was tested to be necessary), and final polymerization at 60 °C for 24 h. Blocks were attached to the microtome holder with commercial two-component epoxy glue and serial-sectioned at 70 μm using VT1000 S vibratome (Leica). Sections were unfolded at 70 °C, mounted in a glycerol and examined using Provis AX70 (Olympus Corporation) light microscope equipped with a digital camera UC90.

Spikelets for SEM were trimmed to expose the basal part of a glume or lemma/palea, or the adaxial side of lemma or palea. Next the specimens were critical-point dried using CPD 7501 dryer (Polaron), attached to the holders and gold sputtered in sputter coater JFC-1300 (JEOL). They were examined in the Analytical Centre of WULS using Quanta 200 (FEI Company) environmental scanning electron microscope operating at 20 kV under high vacuum. The images were saved as tiff files at 2048 × 1886 pixels resolution.

### Lemma kinetics

The tests were performed in two replicate experiments done on May 15–31, 2018 and May 20–June 10, 2019. Just before the start of flowering, the plants were cut off at the base of the stem, submerged with the basal ends of the stems in a container of water and placed in a plant grow box, under continuous light source (HPS Phytolite 600 W lamp, photon flux1045 lmol m-2 s-1, luminous flux 100 klm) at 21 °C. Measurements of the lemmas opening kinetics were based on films recorded using the time-lapse movie method (Ricoh GR cameras, Japan) and analyzed in the Tracker program (https://www.cabrillo.edu/dbrown/tracker/) based on the Open Source Physics (OSP) Java framework. Fifteen spikelets of each of the two cultivars were used in the observations of lemmas opening kinetic in each experiment. Lemmas potential deformations during the flowering process were examined using a 3D image correlation method based on two cameras in a stereoscopic setup connected to a stereoscopic microscope. Such a technique gives the opportunity to perform measurements of 3D deformation and strain of specimens. Each camera provides a 2D view and software, through a correlation algorithm, forms a 3D image combining both camera views from different angles [42]. Present measurements of the wheat lemma deformation were made using the Dantec Q-400 system based on the Istra 4D software module with 5 MPix optics connected to the Leica M125 stereoscopic microscope. Activated carbon particles were sprayed on the lemma to create a stochastic pattern to the surface. The changes of this pattern due to deformation or movement of the object are recorded by the cameras. These images were automatically analyzed with special high accuracy correlation algorithms. As the result a set of data was generated containing the start contour of the object at beginning of the measurement and the three dimensional displacement vector of each object point due to the object deformation. In order to prevent possible other movements, not related to the lemma opening process each spikelet was inserted in a floristic sponge. The deformation measurements were made in 2019 on two replicate lemmas of each of the two cultivars.

### Statistical analyses

The data are presented as means with standard deviations (SD). Statistical analyses was based on a t-test for independent groups used to compare the means of analyzed pollen data between cultivars and between years within cultivars. The pair-wise U Mann-Whitney’s non-parametric test was applied for the number of anthers per spike and the proportion of extruded anthers as the data did not fit t-test assumptions. The level of statistical significance for all the analyses was *P* = 0.05. The analyses were performed using Statistica ver. 13.3 software (Statsoft Polska, Kraków, Poland).

## Supplementary Information

**Additional file 1: Table S1.** Diurnal pattern of anther extrusion in two wheat cultivars. Original data from two experiments in 2018 and 2019 are summarized in Fig. 1. **Table S2.** Proportion of extruded anthers in two wheat cultivars. Original data from two experiments in 2018 and 2019 are summarized in Fig. 2. **Table S3.** Number of pollen grains per anther in two wheat cultivars. Original data from two experiments in 2018 and 2019 are summarized in Table 1. **Table S3.** Number of pollen grains per anther in two wheat cultivars. Original data from two experiments in 2018 and 2019 are summarized in Table 1. **Table S4.** Components of pollen production (columns C, D,) and the number of pollen grains (E-H) in two wheat cultivars. Original data from two experiments in 2018 and 2019., Data from columns C, D and columns E-H are summarized in Table 1 and Fig. 3, respectively. **Table S5.** Spatial displacement (mm) of the opening lemmas. Results of two experiments during flowering of Dacanto and Piko wheat cultivars carried in 2018 and 2019. Data of 15 replicate lemmas obtained from the time-lapse imaging. The data are summarized in Fig. 7. **Table S6.** Speed of spatial displacement (mm/min) of the opening lemmas. Results of two experiments during flowering of Dacanto and Piko wheat cultivars carried in 2018 and 2019. Data of 15 replicate lemmas obtained from the time-lapse imaging. The data are summarized in Fig. 7.

## Data Availability

The materials used during the current study are included in the Supplementary Information: Additional file 1. Tables S1-S6.

## Abbreviations

LM: Light microscopy
SEM: Scanning electron microscopy
ULS: University of Life Sciences in Lublin
WULS: Warsaw University of Life Sciences - SGGW

## References

[1] Le Corff J (1993). Effects of light and nutrient availability on chasmogamy and cleistogamy in an understory tropical herb, *Calathea micans* (Marantaceae). Am J Bot.

[2] De Vries A (1971). Flowering biology of wheat, particularly in view of hybrid seed production—a review. Euphytica..

[3] Waddington SR, Cartwright PM, Wall PC (1993). A qualitative scale of spike initial and pistil development in barley and wheat. Ann Bot.

[4] Langer S, Longin C, Würschum T (2014). Phenotypic evaluation of floral and flowering traits with relevance for hybrid breeding in wheat (*Triticum aestivum* L.). PBI..

[5] Longin CF, Mühleisen J, Maurer HP, Zhang H, Gowda M, Reif JC (2012). Hybrid breeding in autogamous cereals. Theor Appl Genet.

[6] Mette MF, Gils M, Longin CFH, Reif JC, Ogihara Y, Takumi S, Handa H (2015). Hybrid breeding in wheat. Advances in Wheat Genetics: From Genome to Field.

[7] Boeven PH, Longin CF, Leiser WL, Kollers S, Ebmeyer E, Würschum T (2016). Genetic architecture of male floral traits required for hybrid wheat breeding. Theor Appl Genet.

[8] Gupta PK, Balyan HS, Gahlaut V, Saripalli G, Pal B, Basnet BR, Joshi AK (2019). Hybrid wheat: past, present and future. Theor Appl Genet.

[9] Gowda M, Longin CFH, Lein V, Reif JC (2012). Relevance of specific versus general combining ability in winter wheat. Crop Sci.

[10] Whitford R, Fleury D, Reif J, Garcia M, Okada T, Korzun V, Langridge P (2013). Hybrid breeding in wheat: technologies to improve hybrid wheat seed production. J Exp Bot.

[11] Dixon LE, Bencivenga S, Boden SA (2018). A new opening for wheat seed production. JXB..

[12] Liu H, Zhang G, Wang J, Li J, Song Y, Qiao L, Niu N, Wang J, Ma S, Li L (2018). Chemical hybridizing agent SQ-1-induced male sterility in *Triticum aestivum* L.: a comparative analysis of the anther proteome. BMC Plant Biol.

[13] Percival J (1921). The wheat plant.

[14] Yoshida H (2012). Is the lodicule a petal: molecular evidence?. Plant Sci.

[15] De Vries A (1974). Some aspects of cross-pollination in wheat (*Triticum aesticum* L.). another length and number of pollen grains per anther. Euphytica..

[16] Zee S, O’Brien T (1971). The vascular tissue of the lodicules of wheat. Aust J Biol Sci.

[17] Frankel R, Galun E (1977). Pollination mechanisms, reproduction and plant breeding.

[18] Virmani SS (1994). Heterosis and hybrid rice breeding.

[19] Heslop-Harrison Y, Heslop-Harrison JS (1996). Lodicule function and filament extension in the grasses: potassium ion movement and tissue specialization. Ann Bot.

[20] Kirby EJM, Fellowes G, Appleyard M (1983). An thesis in winter barley. Annual report—Plant Breeding Institute.

[21] Pickett AA (1993). Hybrid wheat - results and problems.

[22] Okada T, Ridma JEA, Jayasinghe M, Nansamba M, Baes M, Warner P, Kouidri A, Correia D, Nguyen VY, Whitford R, Baumann U (2018). Unfertilized ovary pushes wheat flower open for cross-pollination. J Exp Bot.

[23] Barrett SC (2002). The evolution of plant sexual diversity. Nat Rev Genet.

[24] Van Doorn WG, Van Meeteren U (2003). Flower opening and closure: a review. J Exp Bot.

[25] Ostergaard L (2009). Fruit Development and Seed Dispersal (L. Ostergaard, Ed.).

[26] Llorens C, Argentina M, Rojas N, Westbrook J, Dumais J, Noblin X (2016). The fern cavitation catapult: mechanism and design principles. J R Soc.

[27] De Vries A (1972). Some aspects of cross-pollination in wheat (*Triticum aestivum L.)* 1. Pollen concentration in the field as influenced by variety, diurnal pattern, weather conditions and level as compared to the height of the pollen donor. Euphytica.

[28] Kempe K, Boudichevskaia A, Jerchel R, Pescianschi D, Schmidt R, Kirchhoff M, Schachschneider R, Gils M (2013). Quantitative assessment of wheat pollen shed by digital image analysis of trapped airborne pollen grains. ACST..

[29] Beri SM, Anand SC (1971). Factors affecting pollen shedding capacity in wheat. Euphytica..

[30] Arber A (1934). The reproductive shoot in grasses – structure and anthesis. The Gramineae. A Study of Cereal, Bamboo and Grass.

[31] Beuzamy L, Nakayama N, Boudaoud A (2014). Flowers under pressure: ins and outs of turgor regulation in development. Ann Bot.

[32] Pandey B, Sharma P, Pandey DM, Sharma I, Chatrath R (2013). Identification of new aquaporin genes and single nucleotide polymorphism in bread wheat. Evol Bioinformatics Online.

[33] Singh RB, Sindhu JS (1974). Pollen production and shedding in male fertility restorer lines of wheat. Wheat Inf Serv.

[34] Antoń S, Denisow B, Milaniuk K (2014). Flowering, pollen production and insect visitation in two *Aconitum* species (Ranunculaceae). Acta Agrobot.

[35] Bhattacharya K, Datta BK (1992). Anthesis and pollen release: anthesis and pollen release of some plants of West Bengal, India. Grana.

[36] Hedhly A, Hormaza JI, Herrero M (2009). Global warming and sexual plant reproduction. Trends Plant Sci.

[37] Browne RG, Iacuone S, Li SF, Dolferus R, Parish RW (2018). Another morphological development and stage determination in *Triticum aestivum*. Front Plant Sci.

[38] Ji X, Shiran B, Wan J, Lewis DC, Jenkins CLD, Condon AG, Richards RA, Dolferus R (2010). Importance of pre-anthesis anther sink strength for maintenance of grain number during reproductive stage water stress in wheat. Plant Cell Environ.

[39] Monneveux P, Pastenes C, Reynolds MP (2003). Limitations to photosynthesis under light and heat stress in three high-yielding wheat genotypes. J Plant Physiol.

[40] Schwab B, Hülskamp M (2010). Quick and easy fixation of plant tissues for scanning electron microscopy (SEM). Cold Spring Harbor Protoc.

[41] Maácz GJ, Vágás E (1961). A new method for staining of cellulose and lignified cell-walls. Mikroskopie..

[42] Rusin T, Kopernik M (2016). Characterization of biocompatible materials using stereo microscope 3D digital image correlation. Adv Eng Mater.

