# Supplementary material for: Spikelet movements, anther extrusion and pollen production in wheat cultivars with contrasting tendencies to cleistogamy
Source: BMC Plant Biol. 2021 Mar 16;21:136. doi: 10.1186/s12870-021-02917-7 (PMC7970976; doi:10.1186/s12870-021-02917-7)
Supplement: Supplementary file 4 — Additional file 1: Table S1. Diurnal pattern of anther extrusion in two wheat cultivars. Original data from two experiments in 2018 and 2019 are summarized in Fig. 1. Table S2. Proportion of extruded anthers in two wheat cultivars. Original data from two experiments in 2018 and 2019 are summarized in Fig. 2. Table S3. Number of pollen grains per anther in two wheat cultivars. Original data from two experiments in 2018 and 2019 are summarized in Table 1. Table S3. Number of pollen grains per anther in two wheat cultivars. Original data from two experiments in 2018 and 2019 are summarized in Table 1. Table S4. Components of pollen production (columns C, D,) and the number of pollen grains (E-H) in two wheat cultivars. Original data from two experiments in 2018 and 2019., Data from columns C, D and columns E-H are summarized in Table 1 and Fig. 3, respectively. Table S5. Spatial displacement (mm) of the opening lemmas. Results of two experiments during flowering of Dacanto and Piko wheat cultivars carried in 2018 and 2019. Data of 15 replicate lemmas obtained from the time-lapse imaging. The data are summarized in Fig. 7. Table S6. Speed of spatial displacement (mm/min) of the opening lemmas. Results of two experiments during flowering of Dacanto and Piko wheat cultivars carried in 2018 and 2019. Data of 15 replicate lemmas obtained from the time-lapse imaging. The data are summarized in Fig. 7. [file 12870_2021_2917_MOESM1_ESM.docx]

**SUPPLEMENTARY TITLE PAGE**

**TITLE:**

**Spikelet movements, anther extrusion and pollen production in wheat cultivars with contrasting tendencies to cleistogamy**

**AUTHORS:**

Urszula Zajączkowska, Bożena Denisow, Barbara Łotocka, Alicja Dołkin-Lewko, Monika Rakoczy-Trojanowska

**SUPPLEMENTARY INFORMATION**

**Additional file 1. Tables S1-S6**

**Table S1**. Diurnal pattern of anther extrusion in two wheat cultivars. Original data from two experiments in 2018 and 2019 are summarized in Fig. 1.

**Table S2**. Proportion of extruded anthers in two wheat cultivars. Original data from two experiments in 2018 and 2019 are summarized in Fig. 2.

**Table S3**. Number of pollen grains per anther in two wheat cultivars. Original data from two experiments in 2018 and 2019 are summarized in Table 1.

**Table S3**. Number of pollen grains per anther in two wheat cultivars. Original data from two experiments in 2018 and 2019 are summarized in Table 1.

**Table S4**. Components of pollen production (columns C, D,) and the number of pollen grains (E-H) in two wheat cultivars. Original data from two experiments in 2018 and 2019. , Data from columns C, D and columns E-H are summarized in Table 1 and Fig. 3, respectively.

**Table S5**. Spatial displacement (mm) of the opening lemmas. Results of two experiments during flowering of Dacanto and Piko wheat cultivars carried in 2018 and 2019. Data of 15 replicate lemmas obtained from the time-lapse imaging. The data are summarized in Fig. 7

**Table S6**. Speed of spatial displacement (mm/min) of the opening lemmas. Results of two experiments during flowering of Dacanto and Piko wheat cultivars carried in 2018 and 2019. Data of 15 replicate lemmas obtained from the time-lapse imaging. The data are summarized in Fig. 7.

**Additional file 2. Movie 1**

Example of the original time-lapse movie recorded during the lemmas opening in cv. Dacanto.

**Additional file 3. Movie 2**

Example of the original time-lapse movie recorded during the lemmas opening in cv. Piko.

**Additional file 4. Movie 3**

Movie illustrating, the change in the position of the lemma during flowering.

Original records from two cameras connected to a stereoscopic microscope, which were the basis for determining the potential lemma deformation using 3-D image correlation method
